# Analysis of the expression of KIR and HLA-Cw in a Northeast Han population

**DOI:** 10.3892/etm.2012.763

**Published:** 2012-10-25

**Authors:** YU HAN, LING ZHAO, ZHENYU JIANG, NING MA

**Affiliations:** 1 Jilin Blood Center, Changchun, Jilin 130033;; 2 Department of Rheumatology, First Hospital, Jilin Unversity, Changchun 130021, P.R. China

**Keywords:** killer cell immunoglobulin-like receptor, human leukocyte antigen-Cw, ligand, Jilin Han ethnicity

## Abstract

The aim of this study was to investigate the expression of the human leukocyte antigen (HLA)-Cw and killer cell immunoglobulin-like receptor (KIR) genes in a Jilin Han population and to provide a theoretical basis for further studies of their roles in disease. A total of 154 unpaid Jilin Han blood donors were selected and KIR and HLA-Cw genotyping was performed using PCR-SSP. Recognition of HLA-Cw and the corresponding activatory or inhibitory KIR receptor was distinguished according to the identification of HLA-Cw and KIR. In the present study, the expression frequency of HLA-C2^Lys80^+2DL1 was 27.27%, HLA-C1^Asn80^+2DL2/2DL3 was 68.83%, 2DS2+HLA-C1^Asn80^ was 9.74% and 2DS1+HLA-C2^Lys80^ was 9.74%. Of the individuals in the study, 72.08% expressed only KIR2DL1 without HLA-Cw, 21.43% expressed only KIR2DS1 without HLA-Cw^Lys^-KIR2DL1 and 2.60% expressed only KIR2DS2 without HLA-Cw^Asn^-KIR2DL2/L3. In conclusion, the expression of inhibitory HLA-Cw-KIR is higher than the expression of activating HLA-Cw-KIR and approximately 20% of the individuals separately expressed the activated HLA-Cw-KIR in the Jilin Han population in the present study.

## Introduction

Killer cell immunoglobulin-like receptor (KIR), expressed by natural killer (NK) cells and certain T cells, is a type of CD158 glucoprotein (1). Through the recognition of human leukocyte antigen (HLA)-I, KIR regulates the cytoactivity of the effector cell, begins the immunological process and regulates cytokine secretion. KIR gene clusters have extremely complex gene structures gradually formed from the single gene structure of KIR3DX1 (2). The human KIR gene cluster contains fifteen genes and two silencers. The KIR gene cluster encoding the receptor contains protein guide sequences (exons 1 and 2), the extracellular domain (exons 3–5), stem (exon 6), transmembrane domain (exon 7) and intracellular domain (exons 8 and 9). The KIR gene cluster genes are named according to their extracellular domain and intracellular domain structural features. An extracellular domain with two similar immunoglobulin domains would be named 2D, or 3D if it had three similar immunoglobulin domains. KIR genes with longer intracellular domains and transduction inhibitory signals are designated L, while those with shorter intracellular domains and transduction activition signals are designated S. The main ligand of KIR is the HLA-I class antigen. Following the recognition of various HLAs, the KIR receptor determines whether the object belongs to the ‘self’ or not, thus making it extremely important in immune regulation (3,4). The KIRs may be divided into two categories: stimulatory KIRs (sKIRs) and inhibitory KIRs (iKIRs).

The specific receptors of certain inhibitory KIR class HLA-I molecules are already known. The ligand of the inhibitory genes KIR2DL1, -2DL2 and -2DL3 is HLA-C. Based on the amino acid combination at the heavy chain loci 80, HLA-C has been divided into two groups. In the HLA-C1 group, the amino acid of site 80 is lysine, while in the HLA-C2 group it is aspartic acid. The ligand of KIR2DL1 is HLA-C2 and the ligand of KIR2DL2/2DL3 is HLA-C1 (5). KIR2DL1+HLA-C2 combined excitatory and inhibitory signals are stronger than those of KIR2DL2/2DL3+HLA-C1 (6). The ligand of KIR3DL1/S1 is the HLA-B antigen which contains a Bw4 structure. It has been reported that KIR3DL1/S1 is able to combine with the HLA-A antigen, which also contains a Bw4 structure (7). The ligand of KIR3DL2 is HLA-A3 or HLA-A11. The ligand of KIR2DL4 is HLA-G. The ligand of KIR2DS1-5, an activating gene of KIR, may be an HLA class I antigen although there is no direct evidence to support this theory. Individuals with variations in the KIR gene cluster number and type may be divided into the haploid A and B subtypes, according to its haploid configuration. Haploid A contains frame gene KIR2DL1, KIR2DL3, KIR3DL1 suppressor gene, and KIR2DS4 activating gene. Other configurations are collectively referred to as haploid B. Haploid B contains a number of activating genes.

HLA-Cw is distributed widely in human tissue cells, specifically recognized by KIR and involved in the regulation of NK cell function, which is the basis of NK cell recognition of self and non-self molecules. When the same HLA-Cw molecule is combined with different KIRs, it produces different signals to activate or inhibit the killing function of the NK cells (8). Observations have revealed that there are differences between individuals in the combined forms of HLA-Cw and sKIR or iKIR, which not only affect susceptibility to disease (9) but also disease progression (10). In order to understand the method of the recognition of HLA-Cw and KIR in a Chinese population, the KIR and its HLA-Cw ligands of 154 normal Han individuals from Jilin were examined by PCR-SSP and the identification of HLA-Cw and KIR was analyzed, to provide a the theoretical basis for future research concerning its role in disease progression.

## Materials and methods

### Objective of the study

A total of 154 normal specimens were selected from unpaid blood donors from a Jilin Han population. This group contained 84 males and 70 females (no blood relations) aged between 21 and 50 years old. Venous blood (5 ml) was obtained, placed in test tubes containing EDTA anticoagulants and stored in a −80°C refrigerator. The experimental protocol was established according to the guidelines of the Declaration of Helsinki and was approved by Human Ethics Committee of Jilin University. Informed consent was obtained from each subject prior to their inclusion in the study.

### Genomic DNA extraction

Using the TIANamp Blood DNA kit (Tiangen Biotech Beijing Co. Ltd., Beijing, China), genomic DNA was extracted according to the manufacturer’s instructions. The DNA concentration was ≥80 mg/l and the purity was A_260_/_280_ = 1.65–1.90.

### Primer synthesis

A previously published method for synthesizing the amplified KIR genes using PCR-SSP genotyping primers (Table I) was used (11). The internal control primers of reactions 1 to 15 were FGH (5′-GCCTTCCCAACCATTCCCTTA-3′) and RGH (5′-TCACGGATTTCTGTTGTGTTTC-3′). The length of the amplification product was 429 bp; the internal contrast primers of reaction 16 were DRB1-F (5′-TGCCAAGTGGAGCACCCAA-3′) and DRB1-R (5′-GCATCTTGCTCTGTGCAGAT-3′) and the length of the amplification products was 796 bp. The primers of the HLA-Cw gene for PCR-SSP genotyping were in accordance with those of Mandelboim *et al*(5). The primers are shown in Table II. All primers were synthesized by Beijing DingGuo ChangSheng Biotechnology Co., Ltd. (Beijing, China) and verified by BLAST.

### KIR genes and the HLA-Cw PCR-SSP classification

Each KIR gene required two pairs of KIR gene primers (upstream and downstream) plus an internal contrast primer system. The HLA-Cw gene also required upstream and downstream primers and an internal contrast primer system.

To amplify KIR genes by PCR-SSP (12), 1 μl of each primer mix (5 μM) was dispensed into separate wells in 384-well plates. Each sample required two vertical rows of wells but KIR2DS1 and KIR3DP1 required only one well. PCR cocktails were prepared for each sample with a total volume of 132 μl (enough for 33 reactions) as follows: 200 ng DNA, 16.5 μl 10X PCR buffer (final concentration, 1X), 4.95 μl MgCl_2_ (final concentration 1.5mM), 1.32 μl dNTP (final concentration 200 mM) and 0.825 μl *Taq* polymerase. PCR cocktail (4 μl) was added to each primer mix (total PCR volume = 5 μl). The samples were amplified using a programmable thermal cycler with a heated lid using the following parameters: 3 min at 94°C, 5 cycles of 15 sec at 94°C, 15 sec at 65°C and 30 sec at 72°C; 21 cycles of 15 sec at 94°C, 15 sec at 60°C and 30 sec at 72°C; 4 cycles of 15 sec at 94°C, 1 min at 55°C and 2 min at 72°C with a final 7-min extension step at 72°C.

The HLA-Cw genes were amplified using PCR-SSP genotyping (12). The PCR system was as follows: In each reaction, 100 ng of genomic DNA was amplified in 15 μl of 1X PCR master mix [67 mM Tris-HCl, pH 8.8, 16.6 mM (NH_4_)_2_SO_4_, 0.1% Tween-20, 2 mM MgCl_2_, 200 mM dNTP] containing specific (0.1–0.8 μm) and control primers, as well as 0.5 units *Taq* polymerase. PCR conditions were: 2 min at 94°C, 10 cycles of 10 sec at 94°C and 60 sec at 65°C; 20 cycles of 10 sec at 94°C, 50 sec at 61°C and 30 sec at 72°C with a final 10-min extension step at 72°C.

### Gel electrophoresis

The amplification products (5 μl) were electrophoresed on 2% agarose gels with a migration distance of ∼3 cm and stained with ethidium bromide. The amplification was evaluated on a UV transilluminator and photographed.

### Analysis of the recognition of HLA-Cw and KIR

The difference in the method of recognition between various HLA-Cws and KIRs depended on the 80th amino acid residues of the HLA-Cw. If the residue was lysine (Lys; HLA-Cw2^Lys80^), the receptor was KIR2DL1/S1. If the residue was asparagine (Asn; HLA-Cw1^Asn80^), the receptor was KIR2DL2/L3/S2. The amino acid sequence was acquired according to the HLA-Cw classification results. By combining this information with the phenotype of the KIR, separate HLA-Cw and KIR recognition statistics could be analyzed.

### Statistical analysis

The statistical analysis followed that of Martin and Carrington (11) and also included the frequency and genotype frequency of HLA-Cw and KIR in the Han population of Jilin. The KIR gene-frequency (F) was measured by direct counting, the phenotype frequency (pf; %) = total number of positive gene/total nu,ber of research group; the KIR gene frequency was calculated as F = 1−√(1−pf).

## Results

### Electrophoresis of the KIR gene

**Figure 1:**
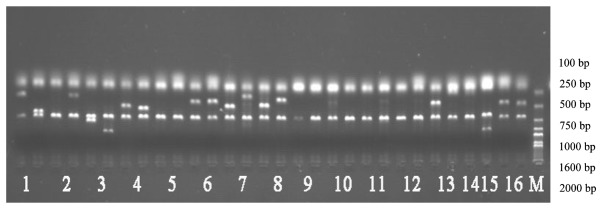
KIR genotyping by PCR-SSP. Lanes 1-16, the PCR-SSP products of KIR2DL1, KIR2DL2, KIR2DL3, KIR2DL4, KIR2DL5, KIR3DL1, KIR3DL2, KIR3DL3, KIR2DS2, KIR2DS3, KIR2DS5, KIR3DS1, KIR2DP1, KIR2DS1 and KIR3DP1, respectively; M, Marker 2000; KIR, killer cell immunoglobulin-like receptor.

Electrophoretic analysis following the conventional amplification of the KIR gene revealed that the size of the target fragment was consistent with the expected results (Fig. 1).

### Electrophoresis of the HLA-Cw gene

**Figure 2:**
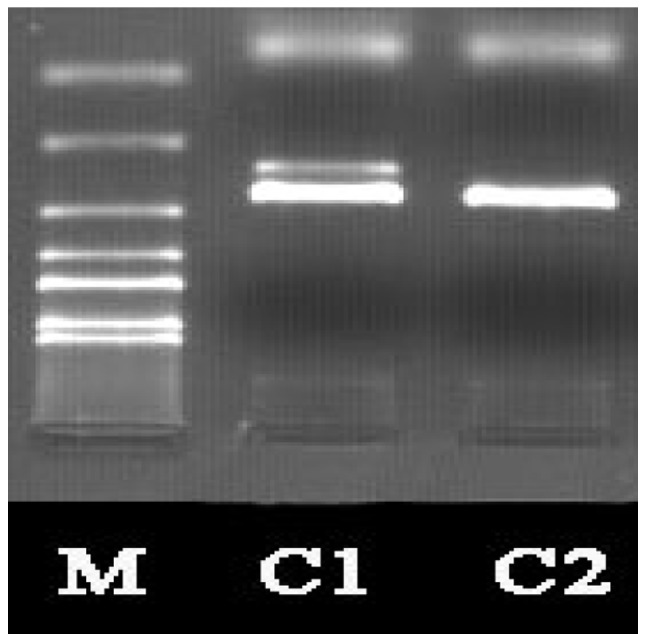
HLA-Cw genotyping by PCR-SSP. M, Marker 2000; C1, PCR-SSP products of HLA-Cw1^Asn^; C2, PCR-SSP products of HLA-Cw2^Lys^; HLA-Cw, human leukocyte antigen-Cw.

Electrophoretic analysis following the conventional amplification of the HLA-Cw gene revealed that the size of the target fragment was consistent with the expected results (Fig. 2).

### Phenotype and genotype frequencies of HLA-Cw and KIR in the Han population in Jilin

The results are shown in Table III.

### Recognition of HLA-C2^Lys^ and KIR

The HLA-C2^Lys^ expression frequency was 27.92%, while 72.08 and 21.43% of individuals expressed only KIR2DL1 or KIR2DS1, respectively, without HLA-C2^Lys^. Table IV shows the distribution of the HLA-Cw KIR activating and inhibitory pairing in the Han population of Jilin. The frequency of HLA-C2^Lys80^+2DL1 was 27.27% and that of 2DS1+HLA-C2^Lys80^ was 9.74%. Of the studied individuals, 21.43% expressed only KIR2DS1 without HLA-C2^Lys^-KIR2DL1.

### Recognition of HLA-C1^Asn^ KIR

The HLA-C1^Asn80^ expression frequency was 79.87%, notably higher than the expression frequency of HLA-C2^Lys80^ which was 27.92%, while 34.42% of individuals expressed only HLA-C1^Asn^ without iKIR or sKIR. Table IV shows the distribution of the HLA-Cw KIR activating and inhibitory pairings in the Han population of Jilin. The frequency of HLA-C1^Asn80^+2DL2/2DL3 was 68.83% and that of 2DS2+HLA-C1^Asn80^ was 9.74%. Of the studied individuals, 2.60% expressed only KIR2DS2 without HLA-C1^Asn^-KIR2DL2/L3.

## Discussion

NK cells recognize virus-infected tissue and tumor cells, then modulate the immune response by killing the target cells through cytotoxic effects or secreting cytokines. NK cells constitute the first barrier of the human immune system (13,14). Immature NK cells must be ‘educated’ by the MHC HLA-Class I molecules, in order to become mature NK cells (15). NK cells adjust their functional status precisely through inhibiting or activating receptors on the membrane surface. In the long-term coevolution of the KIR and the HLA, now highly efficient and accurate receptor-ligand system is formed gradually. (16). Previously, in a study of 477 cases of malaria, Hirayasu *et al*(17) observed that there was a marked correlation between the KIR2DL3 gene and its ligand HLA-C1 in the cerebral malaria patients compared with non-cerebral malaria patients. Further research revealed that the frequency of the combination of the KIR2DL3-HLA-C1 in the malaria high-risk population was maintained at a low level.

Thus, the KIR and HLA combination system is important in regulating NK cell function. Therefore, determining the method of recognition of the HLA-Cw and KIR is particularly important. A total of 154 Han individuals from Jilin were selected. The KIR and HLA-Cw genotyping results revealed that the expression frequency of HLA-C1^Asn80^ was 79.87%, lower than the 97.5% of a Han population in Guangdong (18) and the 76% of a population in Iran (12). The expression frequency of HLA-C2^Lys80^ in the Han population of Jilin was 27.92%, which was equal to that of the Guangdong Han population (18) and lower than the frequency in the Iranian population (12). The frequency of HLA-C1^Asn80^ in the Guangdong Han population was 97.5%, which was significantly higher than that of the Jilin Han population. Compared with other populations, the combination frequency of the KIR receptor and its ligand in the Jilin Han population was essentially the same. The frequency of KIR2DS2+HLA-C1^Asn80^ in the Han population of Jilin was 9.74%, less than that of the Guangdong Han (14%) and Iranian (21.5%) populations. The Jilin Han HLA-C1^Asn80^+KIR2DL2/2DL3 frequency was 68.83%, higher than in the Guangdong Han (41.8%) and Iranian (6.5%) populations. These observations may be due to ethnic and regional differences of the HLA and KIR.

In the 154 Han individuals from Jilin, it was also observed that 9.74% of individuals expressed only the combination of 2DS2+HLA-C1^Asn80^ and 9.74% expressed only the combination of 2DS1+HLA-C2^Lys80^. For these individuals, due to the inhibitory signal being unable to completely block the activation of signal transduction, their NK cells may attack other tissues, resulting in autoimmune diseases (10). However, this has yet to be confirmed in patients through larger sample and clinical experiments.

Martin *et al*(9) suggested that the ligands of sKIRs may not be HLA antigens, but bacterial proteins. The authors observed that the KIR2DL1 and KIR2DL2/L3 frequency was high, up to 100%. Therefore the individual expression of HLA-Cw indicates that a corresponding inhibitory ligand receptor is activated. NK cells with sKIR lacking a corresponding HLA-Cw (for example, KIR2DS1 without HLA-Cw2^Lys^) may be activated by bacterial proteins, resulting in autoimmune diseases. In the current study, 21.43% of individuals were observed to express KIR2DS1 without HLA-Cw2^Lys^ inhibitory receptor ligands. Due to the expression frequency of HLA-Cw1^Asn^ being up to 79.87%, no expression of KIR2DS2 only was observed. However, 2.60% of the population expressed KIR2DS2 but not HLA-Cw1^Asn^ inhibitory receptor ligands. The susceptibility to autoimmune disease of such individuals is worthy of investigation.

## Figures and Tables

**Table I:** Primers used for KIR genotyping by PCR-SSP.

Gene	Primer	Sequence	Primer	Sequence	Size (bp)
2DL1	F1	GTTGGTCAGATGTCATGTTTGAA	R1	GGTCCCTGCCAGGTCTTGCG	146
	F2	TGGACCAAGAGTCTGCAGGA	R2	TGTTGTCTCCCTAGAAGACG	330
2DL2	F1	CTGGCCCACCCAGGTCG	R1	GGACCGATGGAGAAGTTGGCT	173
	F2	GAGGGGGAGGCCCATGAAT	R2	TCGAGTTTGACCACTCGTAT	151
2DL3	F1	CTTCATCGCTGGTGCTG	R1	AGGCTCTTGGTCCATTACAA	550
	F2	TCCTTCATCGCTGGTGCTG	R2	GGCAGGAGACAACTTTGGATCA	800
2DL4	F1	CAGGACAAGCCCTTCTGC	R1	CTGGGTGCCGACCACT	254
	F2	ACCTTCGCTTACAGCCCG	R2	CCTCACCTGTGACAGAAACAG	288
2DS2	F1	TTCTGCACAGAGAGGGGAAGTA	R1	GGGTCACTGGGAGCTGACAA	175
	F2	CGGGCCCCACGGTTT	R2	GGTCACTCGAGTTTGACCACTCA	240
2DS3	F1	TGGCCCACCCAGGTCG	R1	TGAAAACTGATAGGGGGAGTGAGG	242
	F2	CATTGACATGTACCATCTATCCAC	R2	AAGCAGTGGGTCACTTGAC	190
2DS5	F1	TGATGGGGTCTCCAAGGG	R1	TCCAGAGGGTCACTGGGC	126
	F2	ACAGAGAGGGGACGTTTAACC	R2	ATGTCCAGAGGGTCACTGGG	178
3DL1	F1	CGCTGTGGTGCCTCGA	R1	GGTGTGAACCCCGACATG	191
	F2	CCCTGGTGAAATCAGGAGAGAG	R2	TGTAGGTCCCTGCAAGGGCAA	186
3DL2	F1	CAAACCCTTCCTGTCTGCCC	R1	GTGCCGACCACCCAGTGA	211
	F2	CCCATGAACGTAGGCTCCG	R2	CACACGCAGGGCAGGG	130
3DS1	F1	AGCCTGCAGGGAACAGAAG	R1	GCCTGACTGTGGTGCTCG	300
	F2	CCTGGTGAAATCAGGAGAGAG	R2	GTCCCTGCAAGGGCAC	180
3DL3	F1	GTCAGGACAAGCCCTTCCTC	R1	GAGTGTGGGTGTGAACTGCA	232
	F2	TTCTGCACAGAGAGGGGATCA	R2	GAGCCGACAACTCATAGGGTA	165
2DL5	F1	GCGCTGTGGTGCCTCG	R1	GACCACTCAATGGGGGAGC	214
	F2	TGCAGCTCCAGGAGCTCA	R2	GGGTCTGACCACTCATAGGGT	191
2DP1	F1	GTCTGCCTGGCCCAGCT	R1	GTGTGAACCCCGACATCTGTAC	205
	F2	CCATCGGTCCCATGATGG	R2	CACTGGGAGCTGACAACTGATG	89
2DS1	F1	CTTCTCCATCAGTCGCATGAA	R1	AGAGGGTCACTGGGAGCTGAC	102
	F2	CTTCTCCATCAGTCGCATGAG			
2DS4	F1	CTGGCCCTCCCAGGTCA	R1	TCTGTAGGTTCCTGCAAGGACAG	204
	F2	GTTCAGGCAGGAGAGAAT	R2	GTTTGACCACTCGTAGGGAGC	197/219
3DP1	F1	GGTGTGGTAGGAGCCTTAG	R1	GAAAACGGTGTTTCGGAATAC	280
	F2	CGTCACCCTCCCATGATGTA			395

KIR, killer cell immunoglobulin-like receptor; F1/F2, forward primers; R1/R2, reverse primers.

**Table II:** Primers used for HLA-Cw genotyping by PCR-SSP.

	Forward primers		Reverse primers		
Gene	Primer	Sequence	Primer	Sequence	Size (bp)
HLA-C1^Asn80^	F	GAGGTGCCCGCCCGGCGA	R	CGCGCAGGTTCCGCAGGC	332
HLA-C2^Lys80^	F	GAGGTGCCCGCCCGGCGA	R	CGCGCAGTTTCCGCAGGT	332

HLA-Cw, human leukocyte antigen-Cw; Asn, asparagine; Lys, lysine.

**Table III:** Phenotype and genotype frequency of KIR and HLA-Cw in the Jilin Han population.

Gene	Number	Phenotype frequency (%)	Genotype frequency
2DL1	153	99.351	0.919
2DL2	21	13.636	0.071
2DL3	153	99.351	0.919
2DL4	154	100	1
2DL5	49	31.818	0.174
3DL1	143	92.857	0.733
3DL2	154	100	1
3DL3	154	100	1
2DS2	19	12.338	0.064
2DS3	18	11.688	0.06
2DS5	37	24.026	0.128
3DS1	43	27.922	0.151
2DP1	153	99.351	0.919
2DS1	48	31.169	0.17
3DP1*003	76	49.351	0.288
3DP1*001/002/004	153	99.351	0.919
2DS4*001/002	113	73.377	0.484
2DS4*003-007	72	46.753	0.27
HLA-C1^Asn80^	123	79.87	0.551
HLA-C2^Lys80^	43	27.922	0.151

KIR, killer cell immunoglobulin-like receptor; HLA-Cw, human leukocyte antigen-Cw; Asn, asparagine; Lys, lysine.

**Table IV:** The combined frequencies of various compound KIR-HLA genetypes in the Jilin Han population.

iKIR+HLA	Frequency (%)	sKIR+HLA	Frequency (%)
HLA-C1^Asn80^+2DL2/2DL3	68.83	2DS2+HLA-C1^Asn80^	9.74
HLA-C2^Lys80^+2DL1	27.27	2DS1+HLA-C2^Lys80^	9.74

KIR, killer cell immunoglobulin-like receptor; iKIR, inhibitory KIR; sKir, stimulatory KIR; HLA, human leukocyte antigen; Asn, asparagine; Lys, lysine.
